# Sex-specific effects of prenatal undernutrition on resting-state functional connectivity in the human brain at age 68

**DOI:** 10.1016/j.neurobiolaging.2022.01.003

**Published:** 2022-01-22

**Authors:** Amber Boots, Moriah E. Thomason, Claudia Espinoza-Heredia, Patrick J. Pruitt, Jessica S. Damoiseaux, Tessa J. Roseboom, Susanne R. de Rooij

**Affiliations:** a Department of Epidemiology and Data Science, Amsterdam University Medical Centers, University of Amsterdam, Amsterdam, The Netherlands; b Amsterdam Public Health Research Institute, Amsterdam, The Netherlands; c Department of Child and Adolescent Psychiatry, New York University Langone Health, New York, NY, USA; d Department of Population Health, New York University Langone Health, New York, NY, USA; e Neuroscience Institute, New York University Langone Health, New York, NY, USA; f Institute of Gerontology, Wayne State University, Detroit, MI, USA; g Department of Psychology, Wayne State University, Detroit, MI, USA; h Department of Obstetrics and Gynaecology, Amsterdam University Medical Centers, University of Amsterdam, Amsterdam, The Netherlands

**Keywords:** Prenatal undernutrition, Fetal programming, Brain development, Brain aging, fMRI

## Abstract

Prenatal nutrition may significantly impact brain aging. Results from the Dutch Famine Birth Cohort indicated that prenatal undernutrition is negatively associated with cognition, brain volumes, perfusion and structural brain aging in late life, predominantly in men. This study investigates the association between prenatal undernutrition and late-life functional brain network connectivity. In an exploratory resting-state functional magnetic resonance imaging study of 112 participants from the Dutch Famine Birth Cohort, we investigated whether the within- and between-network functional connectivity of the default mode network, salience network and central executive network differ at age 68 in men (N = 49) and women (N = 63) either exposed or unexposed to undernutrition in early gestation. Additionally, we explored sex-specific effects. Compared to unexposed participants, exposed participants revealed multiple clusters of different functional connectivity within and between the three networks studied. Sex-specific analyses suggested a pattern of network desegregation fitting with brain aging in men and a more diffuse pattern of group differences in women. This study demonstrates that associations between prenatal undernutrition and brain network functional connectivity extend late into life.

## 1. Introduction

As humans age, many individuals experience gradual brain aging, whilst others display severe signs of pathological brain aging and clinical symptoms of cognitive decline. Increasing evidence supports the important contribution of environmental factors in these inter-individual differences, especially in early life. In particular, environmental factors during prenatal development may trigger a cascade of processes contributing to individual differences in brain aging. This cascade may involve epigenetic adaptations and prenatal programming of endocrine and metabolistic processes (Cao-Lei et al., 2020; Moreno-Fernandez et al., 2020). Additionally, prenatal environmental factors can affect brain development through the available amount of nutrients as building blocks for the developing fetal brain, thereby impacting future brain functioning and reserve capacity.

Prenatal nutrition influences the structure and function of the brain and may also affect brain aging. Data from the Dutch Famine Birth Cohort (DFBC) study revealed that individuals who had been exposed to undernutrition in early gestation had poorer cognitive performance on a Stroop-like color-word incongruence task at age 58. This was not observed in individuals who had been exposed in mid and late gestation. Additionally, 68-year-old men in the DFBC exposed to famine in early gestation had smaller brain volumes and lower cerebral blood flow in regions related to neurodegeneration (anterior and posterior cingulate cortices) (de Rooij et al., 2016, de Rooij et al., 2019). The lower cerebral blood flow potentially reflected a reduction in blood flow demand due to reduced neuronal activity and/or a reduction in blood flow supply as a result of vascular disease in these regions (de Rooij et al., 2019). Using the Brain Age Gap Estimation (BrainAGE) biomarker of brain structure in the same individuals, it was demonstrated that men who had been exposed to undernutrition in early gestation had higher BrainAGE scores, insinuating premature brain aging (Franke et al., 2018). Overall, these results suggest that undernutrition during early gestation affects cognition, brain structure and perfusion in old age.

In the aging brain, before the appearance of structural changes, a rearrangement of functional brain networks can be observed (Balachandar et al., 2015; Joo et al., 2016). The connectivity within and between functional brain networks reflects the intrinsic functioning of the brain and can be studied with resting-state functional magnetic resonance imaging (fMRI). Age-related neural remodeling is likely to reflect both pathological processes and also adaptations and compensatory mechanisms of the aging brain to mitigate cognitive decline (Damoiseaux, 2017). Studying these functional brain networks may provide an insight into early processes related to brain aging, and help us understand neural processes that underlie cognitive decline.

Alterations in multiple brain networks have been associated with brain aging. Often, these alterations are related to the default mode network (DMN), central executive network (CEN) and salience network (SN). In 2011, Menon et al. (Menon, 2011) introduced a triple network model of functional brain networks, including the DMN for intrinsic processing, the CEN for external processing, and the SN as a switch between these networks. Cognitive decline related to both normal and pathological brain aging has most frequently been associated with alterations in connectivity, metabolism, and perfusion within the DMN (Damoiseaux, 2017; de Vis et al., 2018; Pardo et al., 2007; Reiman et al., 1996; Silverman et al., 2001). Most resting-state functional connectivity (FC) studies describe age-related reductions in DMN connectivity, although both increases and decreases have been observed with increasing age (Bai et al., 2011; Damoiseaux et al., 2008; Geerligs et al., 2015; Ng et al., 2016). Besides alterations in the DMN, studies have reported age-related declines in FC within the SN and CEN, as well as differences in between-network FC (Allen et al., 2011; Archer et al., 2016; Geerligs et al., 2015; Ng et al., 2016; Onoda et al., 2012).

Overall, these available data suggest that altered within- and between-network connectivity of the DMN, CEN and SN may serve as a sensitive, non-invasive biomarker of age-associated brain alterations and cognitive decline. As earlier studies in the DFBC suggest accelerated anatomical brain aging after exposure to prenatal undernutrition, alterations in the functional networks included in the triple network model may also be present in this cohort. Moreover, lower perfusion was observed in the anterior (SN) and posterior (DMN) cingulate cortex after prenatal famine exposure in men compared to unexposed men, increasing our interest in studying the FC of these regions as core regions of the triple network model (de Rooij et al., 2019). By examining the differences in brain network FC between those exposed and unexposed in the DFBC, we may be able to identify earlier and more subtle characteristics of premature brain aging after prenatal undernutrition.

In a study of FC in resting-state fMRI scans of 112 DFBC participants, we investigated the following research question: In men and women exposed or unexposed to undernutrition during early gestation, does the within- and between-network FC of the DMN, SN and CEN differ at age 68? Based on the previous indications of cognitive and brain aging in the DFBC and the abovementioned literature on age-related changes in FC, we hypothesized that undernutrition during early gestation would be associated with lower within-network FC and higher between-network FC in these high-order cognitive networks (Damoiseaux, 2017; Farras-Permanyer et al., 2019; Geerligs et al., 2015; Grady et al., 2016; Huang et al., 2015). Furthermore, we explored sex-specific effects following previously reported sex-specific effects of prenatal undernutrition on the brain in the DFBC (de Rooij et al., 2016).

## 2. Materials and methods

### 2.1. Subjects

#### 2.1.1. The Dutch famine birth cohort

The DFBC is composed of 2414 men and women born as term singletons between November 1, 1943 and February 28, 1947 in the Wilhelmina Gasthuis in Amsterdam, the Netherlands. Inclusion criteria were a singleton birth, minimal pregnancy duration of 259 days and the presence of a medical birth record.

Between December 1944 and April 1945, official daily food rations during the Dutch famine varied between 400 and 800 calories. Rations rose above 1000 calories after May 12, 1945. In the DFBC, a participant was considered to be exposed to prenatal undernutrition when the average daily food ration contained <1000 calories during any 13-week period of gestation. Periods of 16 weeks were defined to group individuals exposed to undernutrition during late, mid and early gestation. Participants exposed to undernutrition during early gestation were born between August 19 and December 8, 1945 (Bleker et al., 2021). Individuals born before January 7, 1945 and conceived after December 8, 1945 were included to act as control groups unexposed to undernutrition during gestation. For the current study, only the control groups born before the famine or conceived after the famine, and those exposed to famine in early gestation were invited.

#### 2.1.2. Study sample

Data for the current MRI study were collected between 2012 and 2013, when participants were 68 years of age. Fifty-four percent of individuals (*n* = 1307) from the initial cohort were eligible (alive with a known current address in the Netherlands). There were no differences in birth characteristics between eligible and non-eligible individuals (birth weight 3357 vs. 3333 g, *p* = 0.22; birth length 32.8 vs. 32.9 cm, *p* = 0.22). See De Rooij et al. (de Rooij et al., 2015) for a detailed description of the selection procedure and inclusion of the current study sample. The study was approved by the local medical ethics committee and carried out according to the Declaration of Helsinki. All participants provided written informed consent.

We invited 151 participants from the DFBC to participate in the 2012 study, consisting of a home visit and an MRI session. Of these 151 participants, nine declined to visit the hospital, eight declined because of scanner anxiety, and 15 had contraindications for MRI scanning. Four participants had missing or incomplete fMRI resting-state data, resulting in a total of 115 participants. See Supplementary Materials for measurement details of covariates.

### 2.2. Magnetic resonance imaging

#### 2.2.1. Data acquisition

MRI scans were acquired using a standardized protocol on a 3.0T Philips Ingenia MRI scanner with a 16-channel dStream Head-Spine coil. We analysed data from T1-weighted 3D magnetization-prepared rapid acquisition gradient echo (MPRAGE: voxel size 1.0 × 1.0 × 1.0 mm, FoV 256 × 256 mm, TR 7.0 ms, TE 3.2 ms, 180 slices, FA 9.0°) and resting-state (180 volumes, 37 slices, voxel size 3.0 × 3.0 × 3.3 mm, FoV 240 × 240 mm, TR 2000 ms, TE 27.0 ms, FA 76.1°, duration 6 minutes) scans. Before the resting-state scan, participants were instructed to remain still and relax without falling asleep. A black screen with a white cross in the center was displayed as a focus point.

#### 2.2.2. Data analysis

##### 2.2.2.1. Seed regions of interest.

As seed regions of interest (ROI), we used the ROIs defined from CONN’s independent component analysis (ICA) of the human connectome project dataset of 497 subjects. Details about the seed ROIs are provided in the Supplementary Methods and in Supplementary Fig. 1.

##### 2.2.2.2. Statistical analysis.

MRI data preprocessing steps are described in the Supplementary Methods. Individual connectivity maps were generated in the CONN toolbox seed-to-voxel analysis pipeline using Fisher’s *Z*-transformed Pearson’s *r* correlation coefficients of the BOLD (Blood-Oxygen-Level-Dependent) time course of each voxel throughout the brain with the mean BOLD time course from each seed. The ROI BOLD time series were extracted from unsmoothed data. Second-level analyses were performed by comparing individual maps of network connectivity between groups using two-sided two-sample t-tests. Individuals exposed to famine in early gestation were compared to individuals prenatally unexposed to famine, in line with approaches used in all previous publications arising from this cohort.

Correction for multiple comparisons was performed using cluster-level correction within binarized masks. We applied a small-volume family-wise error (FWE) corrected threshold of *pFWE* < 0.05 and cluster thresholds were estimated by computing the data’s spatial autocorrelation using the Analysis of Functional NeuroImages toolbox (AFNI, https://afni.nimh.nih.gov) 3dFWHMx software with subsequent Monte Carlo simulations (10,000 iterations) using 3dClustSim. For within-network connectivity, the resulting whole brain connectivity maps for each seed were masked by a combined map of the other ROIs within the network. For between-network connectivity, all ROIs of a network were combined as a single seed and masked with a map of another network of interest (e.g. DMN seed masked with binarized CEN map). Cluster thresholds and sizes are reported at voxel-level primary thresholds of *p* < 0.01 and *p* < 0.05 (*pFWE* < 0.05) to provide a complete overview of our results. In addition, the total numbers of significant voxels within the masked areas at uncorrected primary thresholds of *p* < 0.01 and *p* < 0.05 are reported (Supplementary Fig. 2). This uncorrected total number of voxels was not used to determine significant results, but is reported to provide more information and present a summary overview of results.

These analyses were performed for all subjects combined, and for men and women separately to determine potential sex-specific effects. Post-hoc testing was performed for between-network connectivity to determine the network subregion(s) that contributed most significantly to observed effects. In these analyses, cluster level correction for multiple comparisons was applied using the network subregions as ROIs instead of a combined ROI of the total network.

To determine the effect of extracting the ROI BOLD time series from smoothed versus unsmoothed data, a sensitivity analysis was performed on the significant within-network effects. The same analysis pipeline was executed, now using smooth data for extracting the ROI BOLD time series. These results are presented in Supplementary Tables 1 and 2. Lastly, to determine the size and direction of the connectivity values, we extracted the mean connectivity values between the seed regions and significant clusters by creating binarized masks of significant clusters and performing an eigenvariate analysis in SPM12. An overview of these connectivity values is reported in Supplementary Figs. 3–8.

### 2.3. Data availability

The data that support the findings of this study are available on request from the corresponding author by submitting a formal project outline. The data are not publicly available due to participant privacy restrictions. Code is available upon request from the corresponding author.

## 3. Results

### 3.1. Study group characteristics

Of the 115 included participants, three were excluded based on movement (mean absolute displacement > 2.3 mm). There were no differences in mean absolute displacement between exposed and unexposed participants. Maternal, birth and adult characteristics were similar in exposed and unexposed (born before and conceived after) study groups in the current cohort subsample (Table 1). In sex-specific comparisons, exposed men had a significantly smaller total brain volume (*p* = 0.006) than unexposed men, whereas no group differences were observed in women. Within the unexposed group, we found that the born before and conceived after groups showed differences in age (by definition) and body mass index (BMI), with those being born before the famine having lower BMI than those conceived after the famine.

### 3.2. Functional connectivity outcomes

**Fig. 1.**
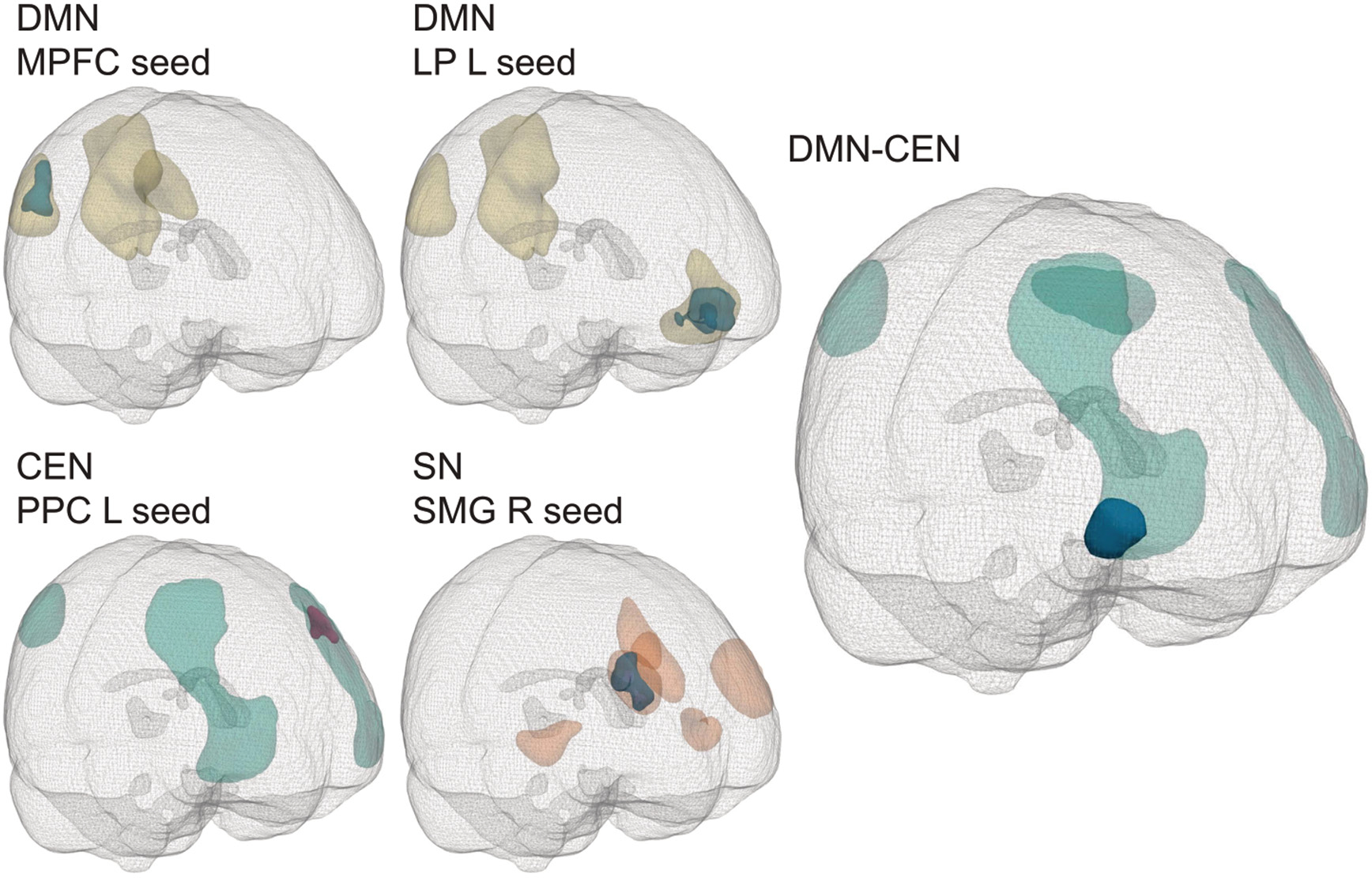
Significant within- and between-network clusters across all participants. Abbreviations: CEN, central executive network; DMN, default mode network; L, left; LP, lateral parietal; MPFC, medial prefrontal cortex; PPC, posterior parietal cortex; R, right; SMG, supramarginal gyrus; SN, salience network. Magenta clusters: exposed > controls, blue clusters: controls > exposed. Light magenta/blue: primary threshold *p* < 0.05, dark magenta/blue: primary threshold *p* < 0.01. Masks are shown in yellow (DMN), orange (SN) and teal (CEN).

For within-network FC across all participants, we observed a cluster of significantly higher positive within-CEN FC between the left PPC and left LPFC for those exposed compared to unexposed participants. Lower positive FC was observed in exposed participants in the DMN (MPFC-right LP) and SN (right SMG-right RPFC). None of these clusters passed the *pFWE* level corrected cluster threshold of *pFWE* < 0.05 at a primary threshold of *p* < 0.01, but did pass the cluster threshold at *p* < 0.05 (Table 2, Fig. 1). In the sensitivity analysis extracting the ROI BOLD time series from smoothed data, the majority of observed effects was similar, although not all observed effects were statistically significant using this approach.

Regarding group differences in between-network FC across all participants, controls had a positive FC, whereas a negative correlation was observed in exposed participants between the DMN and CEN. This cluster was significant at a primary threshold of *p* < 0.01 (*pFWE* < 0.05, Table 3, Fig. 1).

#### 3.2.1. Functional connectivity outcomes in men

**Fig. 2.**
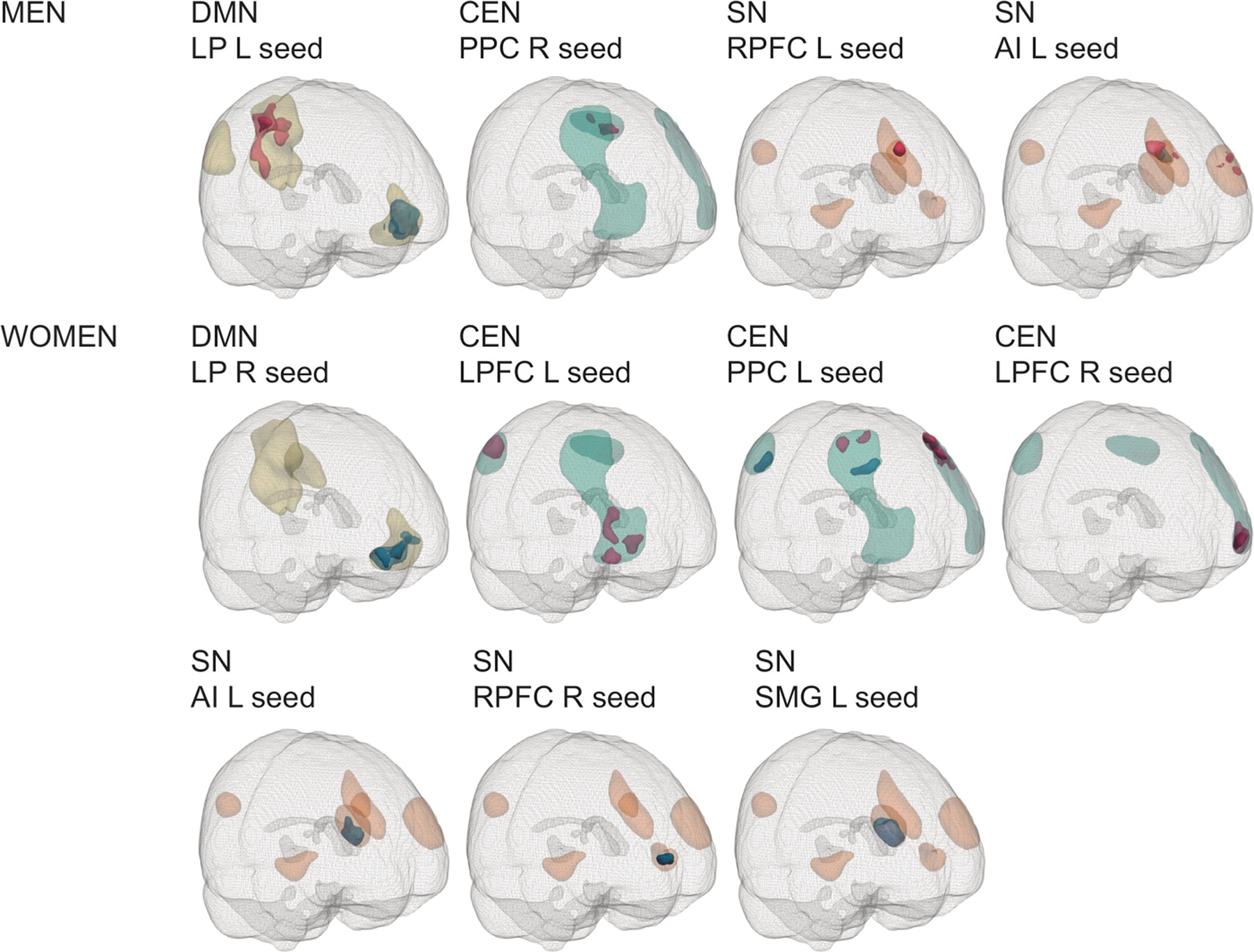
Sex-specific significant within-network clusters. Abbreviations: AI, anterior insula; CEN, central executive network; DMN, default mode network; L, left; LP, lateral parietal; LPFC, lateral prefrontal cortex; PPC, posterior parietal cortex; R, right; RPFC, rostral prefrontal cortex; SMG, supramarginal gyrus; SN, salience network. Magenta clusters: exposed > ceontrols, blue clusters: controls > exposed. Light magenta/blue: primary threshold *p* < 0.05, dark magenta/blue: primary threshold *p* < 0.01. Masks are shown in yellow (DMN), orange (SN) and teal (CEN).

For within-network FC, exposed men had higher positive FC within the DMN (left LP-PCC), SN (left RPFC-ACC, left AI-ACC, left AI-left RPFC) and CEN (right PPC-left PPC) compared to unexposed men. These effects were significant only at a primary threshold of *p* < 0.05, with the exception of the effect between the left RPFC and ACC, which was significant at a primary threshold of *p* < 0.01 and not at *p* < 0.05, and the effect between the left LP and the PCC, which was significant at both primary thresholds (*pFWE* < 0.05). In addition, exposed men had lower positive FC within the DMN (left LP-MPFC, significant at a primary threshold of *p* < 0.05, *pFWE* < 0.05) and SN (left AI-left SMG, significant at a primary threshold of *p* < 0.01 and *p* < 0.05, *pFWE* < 0.05) (Table 4, Fig. 2).

**Fig. 3.**
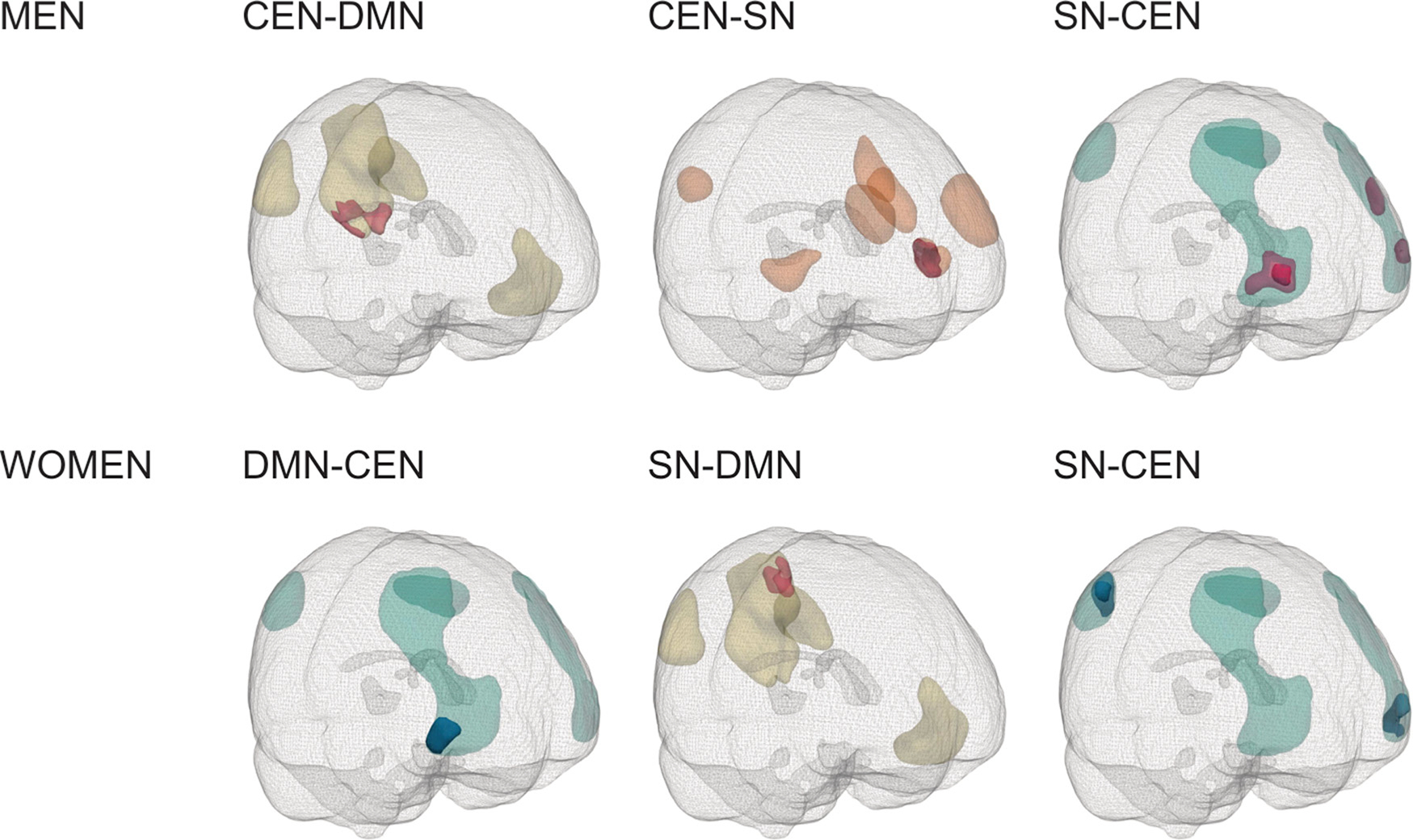
Sex-specific significant between-network clusters. Abbreviations: CEN, central executive network; DMN, default mode network; SN, salience network. Magenta clusters: exposed > controls, blue clusters: controls > exposed. Light magenta/blue: primary threshold *p* < 0.05, dark magenta/blue: primary threshold *p* < 0.01. Masks are shown in yellow (DMN), orange (SN) and teal (CEN).

For between-network FC, compared to unexposed men, we observed clusters of significantly higher FC in exposed men between the CEN and DMN, and between the CEN and SN. Two clusters between the CEN and SN had higher positive FC in exposed men. One cluster between the CEN and DMN and one cluster between the CEN and SN had an anti-correlation in unexposed men, whereas exposed men had a positive FC. All of these clusters were significant at a primary threshold of *p* < 0.05, and one cluster between the SN and CEN was significant at primary threshold *p* < 0.01 (*pFWE* < 0.05, Table 5, Fig. 3).

#### 3.2.2. Functional connectivity outcomes in women

Five different clusters of higher positive FC in exposed women were observed within the CEN (left LPFC-right LPFC, left LPFC-right PPC, left PPC-left LPFC, left PPC-right LPFC) (Fig. 2). Two of these clusters were significant at a primary threshold of *p* < 0.01, and all were significant at *p* < 0.05 (*pFWE* < 0.05). Exposed women had lower positive FC within the DMN (right LP-MPFC), SN (left AI-right RPFC, left SMG-right RPFC) and CEN (left PPC-right LPFC, left PPC-right PPC) than unexposed women.

We observed a cluster of significantly higher positive FC in exposed women between the SN and DMN (significant at a primary threshold of *p* < 0.05, *pFWE* < 0.05), and clusters of significantly lower positive FC between the SN and CEN (two clusters, one significant at a primary threshold of *p* < 0.05, and one at both *p* < 0.01 and *p* < 0.05, *pFWE* < 0.05) (Fig. 3). One cluster between the DMN and CEN was positively correlated in unexposed women and anti-correlated in exposed women (significant at a primary threshold of *p* < 0.01, *pFWE* < 0.05).

#### 3.2.3. Total number of voxels

To present a summary overview of results, Supplementary Fig. 2 shows the total number of voxels that passed a voxel-level *p*-threshold of *p* < 0.01 or *p* < 0.05 for each ROI masked with the combined map of the other ROIs in the network, in the total study group and in men and women separately.

## 4. Discussion

This study aimed to explore intrinsic brain functioning in late life among men and women who had or had not been exposed to famine prenatally. We identified statistically significant differences between exposed and unexposed individuals in both within- and between-network FC at age 68 years. These between-group differences were sex-specific and reflected a mixture of higher and lower FC across brain regions comprising the CEN, DMN and SN.

### 4.1. Interpretation of associations across all participants

Overall, we observed lower FC in the DMN and SN in individuals who had been exposed to famine in early gestation compared to those who had not been exposed. In the aging brain, functional networks are continuously changing in response to neurodegenerative processes to minimize the effects of brain aging on cognitive performance. These adaptations can be in the form of both increases and decreases in FC, believed to reflect neuronal loss and compensatory mechanisms in response to neuronal loss. Reductions in FC within the DMN and SN have previously been reported in relation to (pathological) brain aging (Agosta et al., 2012; Bai et al., 2011; Balachandar et al., 2015; Damoiseaux, 2017; Damoiseaux et al., 2012; Ng et al., 2016; Onoda et al., 2012). Our observation of lower FC in the DMN and SN in exposed individuals compared to unexposed individuals is thus in line with previous studies in our cohort suggesting accelerated cognitive and brain aging (de Rooij et al., 2010; Franke et al., 2018).

In contrast with our hypothesis, we observed higher FC in the CEN in exposed individuals compared to unexposed individuals. Studies have predominantly reported decreased within-CEN FC in relation to brain aging (Allen et al., 2011; Geerligs et al., 2015; Ng et al., 2016), however, both increased and decreased within-CEN FC have been reported in aging populations, with increased FC potentially representing a compensatory mechanism (Agosta et al., 2012; Balachandar et al., 2015; Jockwitz et al., 2017). Without identifying what may be compensated for and establishing an association between increased FC and behavioral outcomes, we can only speculate on whether our results potentially reflect a compensatory mechanism (Cabeza et al., 2018).

Lastly, we observed a negative correlation between regions of the DMN and CEN in individuals who had been exposed to famine in early gestation, in contrast to the positive correlation observed in those who had not been exposed. This observation contradicts our hypothesis of higher between-network FC befitting accelerated brain aging, but does point at alterations in between-network connectivity associated with exposure to famine in early gestation.

### 4.2. Developmental framework

The development of functional brain networks begins *in utero*. A FC component of the DMN with evident coordinated activity between regions of the DMN can already be observed prenatally. At birth, most of the systems eventually developing into adult brain functional networks have manifested (Thomason, 2020; Thomason et al., 2015). Given the rapid development of the brain and its connectivity *in utero*, the brain is especially vulnerable to harmful exposures during this period (Thomason et al., 2015). Recent studies have shown that an exposure during prenatal development can significantly impact developing brain networks *in utero*. For example, Thomason and colleagues (2019) studied FC in the human fetal brain after prenatal lead exposure. They observed diminished age-related increases in cross-hemispheric FC and stronger age-related increases in anterior-posterior FC in exposed fetuses compared to unexposed fetuses (Thomason et al., 2019). Similarly, De Asis-Cruz et al. (De Asis-Cruz et al., 2020) observed both higher and lower fetal brain FC after prenatal maternal anxiety.

Furthermore, numerous studies have investigated the impact of a prenatal exposure on FC of the early postnatal brain. For instance, studies associated prenatal exposure to maternal stress, alcohol, drugs, opioids, and manganese with disruptions of functional network organization in neonates, infants and children (de Water et al., 2018; Donald et al., 2016; Donnici et al., 2021; Fan et al., 2017; Radhakrishnan et al., 2021; Roos et al., 2020; Salzwedel et al., 2015; Scheinost et al., 2016; Wozniak et al., 2011), underlining that exposures during prenatal development affect brain FC in early life.

Such reductions in fetal and infant FC related to prenatal adverse exposures may be at the start of long-term declines in network structure integrity and thereby contribute to cognitive problems later in life (Thomason et al., 2019). Likewise, prenatal undernutrition may be associated with altered FC *in utero*, eventually resulting in altered network connectivity in later life. Other stressful exposures in the prenatal environment may display similar associations, which should be explored.

Although functional brain network organization starts developing after the first trimester, adaptations in the earliest phases of brain development as a result of famine exposure may have been associated with alterations of this developmental trajectory and may have thereby modified the arrangement of functional brain networks *in utero* and thereafter. Whether the group differences in FC observed in the current study have been present since prenatal development or are related to accelerated brain aging associated with prenatal exposure to undernutrition is unclear. Follow-up FC measurements in the cohort at an older age may help answer this question. Of note, many factors throughout life may have additionally impacted the FC of these networks and, thus, direct causality cannot be implied.

### 4.3. Interpretation of effects in sex-specific analysis

In exposed men, we observed clusters of both higher and lower within-network FC, and consistently higher between-network connectivity compared to unexposed men. The observations of lower within-network FC and higher between-network FC fit with our hypothesized pattern of accelerated brain aging in exposed individuals based on network desegregation. In women, the observed group differences reflect both higher and lower within- and between-network connectivity in exposed women compared to unexposed women.

The observed sex-specific outcomes are in line with previous findings in the DFBC which have repeatedly pointed at sex-specific associations of prenatal undernutrition with health outcomes of men and women over time. Associations between prenatal undernutrition and brain size, BrainAGE and brain perfusion at age 68 were primarily observed in men (Bleker et al., 2021). From existing literature, we know that prenatal exposures impact male neurodevelopment significantly more than female neurodevelopment (Bale, 2016). Presumably, this is due to the faster growth rate of male fetuses *in utero* and the protective effects of the female placenta against maternal perturbations during pregnancy (Bale, 2016). Therefore, the male overrepresentation in these DFBC observations associated with brain development may not be surprising. Nevertheless, in the current study, we do observe a convincing list of alterations of within- and between-network FC in exposed women as well. Possibly, exposure to undernutrition during early gestation is more strongly associated with aging-related outcomes in men, resulting in a phenotype befitting an aging population, whereas this association is different in women, showing adaptations in FC that are not necessarily related to aging. Of note, in aging, FC between the DMN and CEN was reported to follow a u-shape, with an initial decrease and thereafter increase (Ng et al., 2016). We observed higher FC between the DMN and CEN in men and lower FC in women after prenatal undernutrition. Speculatively, brain aging in exposed men may have progressed further compared to exposed women, explained by the increased vulnerability of male fetuses to prenatal exposures. Also, this would fit with the potential selection bias of presumably healthier exposed women in the current study as a result of the increased mortality in exposed women observed previously in the DFBC (Bleker et al., 2021). Longitudinal follow-up of FC in this cohort over time is likely to shed further light on these processes.

### 4.4. Strengths and limitations

Study design limitations warrant mention, especially regarding potential bias of the observational study design. Selective fertility should be considered since only the women with sufficient fat reserves did not cease to ovulate and were thus capable of conceiving during the famine. Selection bias may have occurred as a result of selective mortality in early and later life, especially since previous studies in our cohort have shown that exposed women had an increased risk of dying at a younger age. Nevertheless, these forms of bias have most likely contributed to smaller differences between the exposed and unexposed groups and therefore cannot explain the observed group differences. These forms of selection bias cannot be overcome in human cohort studies. Replication in other human cohorts will contribute to a stronger evidence base and should ideally be supplemented with experimental evidence in animal models to address causality. Moreover, many factors between birth and data collection at age 68 may have had an impact on the observed outcomes, limiting the potential of any inferences regarding causality. Nonetheless, our quasi-experimental study design offers the possibility of studying the long-term effects of prenatal undernutrition in an otherwise impossible manner (Bleker et al., 2021). Lastly, increasing the scan duration time might have resulted in more reliable estimates of FC, although increasing scan duration in older participants may also increase motion artefacts. Studies have shown that 5–10 minutes of resting-state data is sufficient for group-level patterns of functional brain organization and group-level differences (Laumann et al., 2015).

Finally, our analysis approach had some implications for the interpretation of the results. First, our sensitivity analysis extracting the ROI BOLD time series data from smoothed versus unsmoothed data underlines the impact of subjective choices in the analysis approach on the outcome of neuroimaging analysis pipelines. The fact that most reported significant clusters were still clearly visible using this different approach strengthens our confidence in the robustness of our results. Further, cluster-extent-based thresholding provides low spatial specificity, especially at higher *p*-value cluster-defining primary thresholds (Woo et al., 2014). Therefore, we can only interpret the significant findings as a general effect within the cluster, and not make any assumptions about the specific location of the effect. Nevertheless, we believe that this was not hindering given that it was not the aim of our study to find prenatal famine related differences in FC at specific locations. As this study was designed as an exploratory study, we did not perform a correction for multiple testing for the independent tests on top of the FDR correction performed in the cluster-wise correction. To prevent overinterpretation of potential false-positive effects, we intentionally did not provide an in-depth discussion of all individual effects, but instead discussed overall within- and between-network effects. Lastly, we did not include a sex-by-group interaction analysis given the small study sample resulting in little power to identify an interaction effect and the large body of evidence of sex-specific associations in our cohort. As a result, we can only report sex-specific associations and cannot make any inferences on potential sex-differences.

Further, a larger sample size would have been preferable. Unfortunately, we were not able to recruit new participants since all eligible cohort members were invited.

### 4.5. Conclusions

We found sex-specific alterations in FC within and between three high-order cognitive functional networks in 68-year-old men and women who had been exposed to famine in early gestation compared to unexposed individuals. This suggests that prenatal nutrition is associated with widespread modifications of intrinsic brain activity in late life. Thereby, this study demonstrates that associations between prenatal undernutrition and brain network functional connectivity extend late into human life. The observed sex-specific effects are in line with previous findings, which have pointed at differential associations between prenatal undernutrition and health outcomes in men and women over time.

Based on the current results, we can only speculate whether these differences have been present since early life, reflect an ongoing process of accelerated brain aging associated with prenatal undernutrition or a combination of early life adaptations and accelerated brain aging. A longitudinal follow-up brain MRI study will enable us to map changes over time, thereby providing further insight on the progression of brain aging in this cohort.

## Supplementary Material

supp

## Figures and Tables

**Table 1:** Maternal, birth and adult characteristics

	N	Born before	Exposed in early gestation	Conceived after	Total	*p*

*General characteristics*						
N		34	40	38	112	
Age (years)	112	68.7 (0.5)^b^	67.4 (0.2)	66.7 (0.4)	67.6 (0.9)	0.22
Women (%)	112	21 (61.8)	22 (55.0)	20 (52.6)	63 (56.3)	0.84
*Maternal and birth characteristics*						
Manual occupation head of family (%)	91	18 (75.0)	23 (63.9)	19 (61.3)	60 (65.9)	0.74
Birth weight (g)	112	3363 (495)	3450 (470)	3384 (501)	3401 (485)	0.43
Head circumference at birth (cm)	111	32.4 (1.3)	32.8 (1.5)	33.0 (1.6)	32.7 (1.5)	0.91
Pregnancy duration (days)	98	285 (11)	287 (11)	286 (14)	286 (12)	0.44
Maternal age at birth (years)	112	28.0 (5.3)	26.3 (6.4)	27.6 (6.4)	27.3 (6.1)	0.19
*Adult characteristics*						
Education (10-point scale)	112	4.8 (2.5)	4.6 (1.8)	4.6 (2.1)	4.6 (2.1)	0.87
Socioeconomic status	112	51 (13)	48 (13)	51 (15)	50 (14)	0.35
Ever smoked (%)	112	20 (58.8)	26 (65.0)	22 (57.9)	68 (60.7)	0.49
BMI (kg/m^2^)	112	27.4 (3.4)^a^	28.4 (5.0)	29.9 (5.7)	28.6 (4.9)	0.72
Hypertension (%)	112	18 (52.9)	18 (45.0)	22 (57.9)	58 (51.8)	0.29
Hypercholesterolemia (%)	111	16 (47.1)	20 (51.3)	15 (39.5)	51 (45.9)	0.41
Diabetes (%)	112	6 (17.6)	9 (22.5)	5 (13.2)	20 (17.9)	0.34
Stroke or TIA (%)	110	1 (2.9)	1 (2.6)	3 (8.1)	5 (4.5)	0.47
HADS anxiety (score)	112	3.6 (2.4)	4.8 (2.9)	4.7 (3.0)	4.4 (2.8)	0.26
HADS depression (score)	112	1.8 (2.0)	2.6 (2.7)	3.1 (4.4)	2.5 (3.2)	0.92
Total brain volume (mm^3^)	112	1016833 (105449)	1017933 (82910)	1043445 (113052)	1026255 (100671)	0.52
BrainAGE (score)	112	−0.8 (4.1)	1.5 (4.6)	0.4 (5.1)	0.4 (4.7)	0.07

Data are displayed as mean (SD) or as frequencies (%). *p*-values for differences between the exposed and combined unexposed (born before and conceived after) groups are based on regression analyses. Superscript represent *p*-values of linear and logistic regression analyses for individuals born before the famine compared to individuals conceived after the famine. Key: BMI, Body Mass Index; HADS, Hospital Anxiety and Depression Scale; TIA, Transient Ischemic Attack.

a *p* < 0.05.

b *p* < 0.001.

**Table 2:** Significant within-network group effects across all participants

Network	Seed region	Subregion effect	Direction of effect	FWE cluster threshold at *p* < 0.01	Cluster size at *p* < 0.01	FWE cluster threshold at *p* < 0.05	Cluster size at *p* < 0.05

CEN	PPC L	LPFC L	EE > HC	44	31	134	139
DMN	MPFC	LP R	HC > EE	88	31	245	260
DMN	LP L	MPFC	HC > EE	105	29	296	421
SN	SMG R	RPFC R	HC > EE	84	50	220	227

Key: CEN, central executive network; DMN, default mode network; EE, early exposed; FWE, Family-Wise Error; HC, healthy controls; L, left; LP, lateral parietal; LPFC, lateral prefrontal cortex; MPFC, medial prefrontal cortex; PPC, posterior parietal cortex; R, right; RPFC, rostral prefrontal cortex; SMG, supramarginal gyrus; SN, salience network.

**Table 3:** Significant between-network group effects across all participants.

Seed network	Mask network	Direction of effect	FWE cluster threshold at *p* < 0.01	Cluster size at *p* < 0.01	FWE cluster threshold at *p* < 0.05	Cluster size at *p* < 0.05	Seed subregion^a^	Mask subregion

DMN	CEN	HC > EE	82	155	223	212	MPFC	LPFC R
							LP R	LPFC R
							PCC	LPFC R

Key: CEN, central executive network; DMN, default mode network; EE, early exposed; FWE, Family-Wise Error; HC, healthy controls; L, left; LP, lateral parietal; LPFC, lateral prefrontal cortex; MPFC, medial prefrontal cortex; PCC, precuneus cortex; R, right.

a Seed subregions were determined in post-hoc analyses using the ‘seed network’ subregions as seeds masked with the ‘mask network’.

**Table 4:** Sex-specific significant within-network group effects.

Network	Seed region	Subregion effect	Direction of effect	FWE cluster threshold at *p* < 0.01	Cluster size at *p* < 0.01	FWE cluster threshold at *p* < 0.05	Cluster size at *p* < 0.05

**Men**							
DMN	LP L	PCC	EE > HC	87	131	245	895
SN	RPFC L	ACC	EE > HC	64	71	197	160
SN	AI L	ACC	EE > HC	38	31	96	132
SN	AI L	RPFC L	EE > HC	38	1	96	102
CEN	PPC R	PPC L	EE > HC	47	2	112	138
DMN	LP L	MPFC	HC > EE	87	67	245	595
SN	AI L	SMG L	HC > EE	38	49	96	110
**Women**							
CEN	LPFC L	LPFC R	EE > HC	56	20	186	235
CEN	LPFC L	PPC R	EE > HC	56	22	186	188
CEN	PPC L^a^	LPFC L	EE > HC	35	60, 73	88	347
CEN	PPC L	LPFC R	EE > HC	35	7	88	88
CEN	LPFC R	LPFC L	EE > HC	60	91	159	251
DMN	LP R	MPFC	HC > EE	89	128	289	477
SN	AI L	RPFC R	HC > EE	56	12	161	165
SN	RPFC R	AI L	HC > EE	75	100	222	200
SN	SMG L	RPFC R	HC > EE	63	55	155	315
CEN	PPC L	LPFC R	HC > EE	35	13	88	91
CEN	PPC L	PPC R	HC > EE	35	1	88	100

Key: ACC, anterior cingulate cortex; AI, anterior insula; CEN, central executive network; DMN, default mode network; EE, early exposed; FWE, Family-Wise Error; HC, healthy controls; L, left; LP, lateral parietal; MPFC, medial prefrontal cortex; PCC, precuneus cortex; PPC, posterior parietal cortex; R, right; RPFC, rostral prefrontal cortex; SMG, supramarginal gyrus; SN, salience network.

a Two significant clusters were identified between these regions.

**Table 5:** Sex-specific significant between-network group effects

Seed network	Mask network	Direction of effect	FWE cluster threshold at *p* < 0.01	Cluster size at *p* < 0.01	FWE cluster threshold at *p* < 0.05	Cluster size at *p* < 0.05	Seed subregion^a^	Mask subregion

**Men**								
CEN	DMN	EE > HC	86	21	245	312	LPFC L	PCC
CEN	SN	EE > HC	92	83	232	291	LPFC L	AI L
							LPFC R	AI L
SN	CEN	EE > HC	39	44	99	169	ACC	LPFC R
							AI L	LPFC R
							AI R	LPFC R
							RPFC L	LPFC R
			39	25, 11	99	126, 119	AI L^b^	LPFC L
			39	11	99	119	AI R	LPFC L
**Women**								
SN	DMN	EE > HC	103	16	289	334	RPFC R	PCC
DMN	CEN	HC > EE	80	127	207	202	MPFC	LPFC R
							LP L	LPFC R
							LP R	LPFC R
							PCC	LPFC R
SN	CEN	HC > EE	50	56	135	183	AI R	PPC R
							SMG L	PPC R
			50	30	135	196	ACC	LPFC L
							SMG L	LPFC L
							SMG R	LPFC L

Key: ACC, anterior cingulate cortex; AI, anterior insula; CEN, central executive network; DMN, default mode network; EE, early exposed; FWE, Family-Wise Error; HC, healthy controls; L, left; LP, lateral parietal; LPFC, lateral prefrontal cortex; MPFC, medial prefrontal cortex; PCC, precuneus cortex; PPC, posterior parietal cortex; R, right; RPFC, rostral prefrontal cortex; SMG, supramarginal gyrus; SN, salience network.

a Seed subregions were determined in post-hoc analyses using the ‘seed network’ subregions as seeds masked with the ‘mask network’.

b Two significant clusters were identified between these regions.

## Abbreviations:

AFNI: Analysis of Functional NeuroImages
BOLD: Blood-Oxygen-Level-Dependent
BrainAGE: Brain Age Gap Estimation
CEN: Central Executive Network
DFBC: Dutch Famine Birth Cohort
DMN: Default Mode Network
FC: Functional Connectivity
FWE: Family-Wise Error
HCP: Human Connectome Project
ICA: Independent Component Analysis
ROI: Region of Interest
SN: Salience Network
BMI: Body Mass Index
TIA: Transient Ischemic Attack
HADS: Hospital Anxiety and Depression Scale

## References

[R1] Agosta F, Pievani M, Geroldi C, Copetti M, Frisoni GB, Filippi M, 2012. Resting state fMRI in Alzheimer’s disease: beyond the default mode network. Neurobiol Aging 33 (8), 1564–1578. 21813210 10.1016/j.neurobiolaging.2011.06.007

[R2] Allen EA, Erhardt EB, Damaraju E, Gruner W, Segall JM, Silva RF, Havlicek M, Rachakonda S, Fries J, Kalyanam R, Michael AM, Caprihan A, Turner JA, Eichele T, Adelsheim S, Bryan AD, Bustillo J, Clark VP, Feldstein Ewing SW, Filbey F, Ford CC, Hutchison K, Jung RE, Kiehl KA, Kodituwakku P, Komesu YM, Mayer AR, Pearlson GD, Phillips JP, Sadek JR, Stevens M, Teuscher U, Thoma RJ, Calhoun VD, 2011. A baseline for the multivariate comparison of resting-state networks. Front Syst Neurosci 5, 2. 21442040 10.3389/fnsys.2011.00002 PMC3051178

[R3] Archer JA, Lee A, Qiu A, Chen SH, 2016. A comprehensive analysis of connectivity and aging over the adult life span. Brain Connect 6 (2), 169–185. 26652914 10.1089/brain.2015.0345

[R4] Bai F, Watson DR, Shi Y, Wang Y, Yue C, Yuhuan Teng, Wu D, Yuan Y, Zhang Z, 2011. Specifically progressive deficits of brain functional marker in amnestic type mild cognitive impairment. PLoS One 6 (9), e24271. 21935394 10.1371/journal.pone.0024271 PMC3174167

[R5] Balachandar R, John JP, Saini J, Kumar KJ, Joshi H, Sadanand S, Aiyappan S, Sivakumar PT, Loganathan S, Varghese M, Bharath S, 2015. A study of structural and functional connectivity in early Alzheimer’s disease using rest fMRI and diffusion tensor imaging. Int J Geriatr Psychiatry 30 (5), 497–504. 24990445 10.1002/gps.4168

[R6] Bale TL, 2016. The placenta and neurodevelopment: sex differences in prenatal vulnerability. Dialogues Clin Neurosci 18 (4), 459–464. 28179817 10.31887/DCNS.2016.18.4/tbale PMC5286731

[R7] Bleker LS, de Rooij SR, Painter RC, Ravelli AC, Roseboom TJ, 2021. Cohort profile: the Dutch famine birth cohort (DFBC)- a prospective birth cohort study in the Netherlands. BMJ Open 11 (3), e042078. 10.1136/bmjopen-2020-042078 PMC7934722 33664071

[R8] Cabeza R, Albert M, Belleville S, Craik FIM, Duarte A, Grady CL, Lindenberger U, Nyberg L, Park DC, Reuter-Lorenz PA, Rugg MD, Steffener J, Rajah MN, 2018. Maintenance, reserve and compensation: the cognitive neuroscience of healthy ageing. Nat Rev Neurosci 19 (11), 701–710. 30305711 10.1038/s41583-018-0068-2 PMC6472256

[R9] Cao-Lei L, de Rooij SR, King S, Matthews SG, Metz GAS, Roseboom TJ, Szyf M, 2020. Prenatal stress and epigenetics. Neurosci Biobehav Rev 117, 198–210. 28528960 10.1016/j.neubiorev.2017.05.016

[R10] Damoiseaux JS, 2017. Effects of aging on functional and structural brain connectivity. Neuroimage 160, 32–40. 28159687 10.1016/j.neuroimage.2017.01.077

[R11] Damoiseaux JS, Beckmann CF, Arigita EJ, Barkhof F, Scheltens P, Stam CJ, Smith SM, Rombouts SA, 2008. Reduced resting-state brain activity in the “default network” in normal aging. Cereb Cortex 18 (8), 1856–1864. 18063564 10.1093/cercor/bhm207

[R12] Damoiseaux JS, Prater KE, Miller BL, Greicius MD, 2012. Functional connectivity tracks clinical deterioration in Alzheimer’s disease. Neurobiol Aging 33 (4), 828.e819–828.e830. 10.1016/j.neurobiolaging.2011.06.024 PMC3218226 21840627

[R13] De Asis-Cruz J, Krishnamurthy D, Zhao L, Kapse K, Vezina G, Andescavage N, Quistorff J, Lopez C, Limperopoulos C, 2020. Association of prenatal maternal anxiety with fetal regional brain connectivity. JAMA Netw Open 3 (12), e2022349. 33284334 10.1001/jamanetworkopen.2020.22349 PMC12549102

[R14] de Rooij SR, Caan MW, Swaab DF, Nederveen AJ, Majoie CB, Schwab M, Painter RC, Roseboom TJ, 2016. Prenatal famine exposure has sex-specific effects on brain size. Brain 139 (Pt 8), 2136–2142. 27401522 10.1093/brain/aww132

[R15] de Rooij SR, Mutsaerts H, Petr J, Asllani I, Caan MWA, Groot P, Nederveen AJ, Schwab M, Roseboom TJ, 2019. Late-life brain perfusion after prenatal famine exposure. Neurobiol. Aging 82, 1–9. 31376728 10.1016/j.neurobiolaging.2019.06.012

[R16] de Rooij SR, van Pelt AM, Ozanne SE, Korver CM, van Daalen SK, Painter RC, Schwab M, Viegas MH, Roseboom TJ, 2015. Prenatal undernutrition and leukocyte telomere length in late adulthood: the Dutch famine birth cohort study. Am J Clin Nutr 102 (3), 655–660. 26178721 10.3945/ajcn.115.112326

[R17] de Rooij SR, Wouters H, Yonker JE, Painter RC, Roseboom TJ, 2010. Prenatal undernutrition and cognitive function in late adulthood. Proc Natl Acad Sci U S A 107 (39), 16881–16886. 20837515 10.1073/pnas.1009459107 PMC2947913

[R18] de Vis JB, Peng SL, Chen X, Li Y, Liu P, Sur S, Rodrigue KM, Park DC, Lu H, 2018. Arterial-spin-labeling (ASL) perfusion MRI predicts cognitive function in elderly individuals: a 4-year longitudinal study. J Magn Reson Imaging 48 (2), 449–458. 29292540 10.1002/jmri.25938 PMC6028323

[R19] de Water E, Proal E, Wang V, Medina SM, Schnaas L, Téllez-Rojo MM, Wright RO, Tang CY, Horton MK, 2018. Prenatal manganese exposure and intrinsic functional connectivity of emotional brain areas in children. Neurotoxicology 64, 85–93. 28610744 10.1016/j.neuro.2017.06.006 PMC5723568

[R20] Donald KA, Ipser JC, Howells FM, Roos A, Fouche JP, Riley EP, Koen N, Woods RP, Biswal B, Zar HJ, Narr KL, Stein DJ, 2016. Interhemispheric functional brain connectivity in neonates with prenatal alcohol exposure: preliminary findings. Alcohol Clin Exp Res 40 (1), 113–121. 26727529 10.1111/acer.12930 PMC6556616

[R21] Donnici C, Long X, Dewey D, Letourneau N, Landman B, Huo Y, Lebel C, 2021. Prenatal and postnatal maternal anxiety and amygdala structure and function in young children. Sci Rep 11 (1), 4019. 33597557 10.1038/s41598-021-83249-2 PMC7889894

[R22] Fan J, Taylor PA, Jacobson SW, Molteno CD, Gohel S, Biswal BB, Jacobson JL, Meintjes EM, 2017. Localized reductions in resting-state functional connectivity in children with prenatal alcohol exposure. Hum Brain Mapp 38 (10), 5217–5233. 28734059 10.1002/hbm.23726 PMC6377933

[R23] Farras-Permanyer L, Mancho-Fora N, Montalà-Flaquer M, Bartrés-Faz D, Vaqué-Alcázar L, Peró-Cebollero M, Guàrdia-Olmos J, 2019. Age-related changes in resting-state functional connectivity in older adults. Neural Regen Res 14 (9), 1544–1555. 31089053 10.4103/1673-5374.255976 PMC6557095

[R24] Franke K, Gaser C, Roseboom TJ, Schwab M, de Rooij SR, 2018. Premature brain aging in humans exposed to maternal nutrient restriction during early gestation. NeuroImage 173, 460–471. 29074280 10.1016/j.neuroimage.2017.10.047

[R25] Geerligs L, Renken RJ, Saliasi E, Maurits NM, Lorist MM, 2015. A brain-wide study of age-related changes in functional connectivity. Cereb Cortex 25 (7), 1987–1999. 24532319 10.1093/cercor/bhu012

[R26] Grady C, Sarraf S, Saverino C, Campbell K, 2016. Age differences in the functional interactions among the default, frontoparietal control, and dorsal attention networks. Neurobiol Aging 41, 159–172. 27103529 10.1016/j.neurobiolaging.2016.02.020

[R27] Huang CC, Hsieh WJ, Lee PL, Peng LN, Liu LK, Lee WJ, Huang JK, Chen LK, Lin CP, 2015. Age-related changes in resting-state networks of a large sample size of healthy elderly. CNS Neurosci Ther 21 (10), 817–825. 25864728 10.1111/cns.12396 PMC6493082

[R28] Jockwitz C, Caspers S, Lux S, Eickhoff SB, Jütten K, Lenzen S, Moebus S, Pundt N, Reid A, Hoffstaedter F, Jöckel KH, Erbel R, Cichon S, Nöthen MM, Shah NJ, Zilles K, Amunts K, 2017. Influence of age and cognitive performance on resting-state brain networks of older adults in a population-based cohort. Cortex 89, 28–44. 28192723 10.1016/j.cortex.2017.01.008

[R29] Joo SH, Lim HK, Lee CU, 2016. Three large-scale functional brain networks from resting-state functional MRI in subjects with different levels of cognitive impairment. Psychiatry Investig 13 (1), 1–7. 10.4306/pi.2016.13.1.1 PMC4701672 26766941

[R30] Laumann TO, Gordon EM, Adeyemo B, Snyder AZ, Joo SJ, Chen MY, Gilmore AW, McDermott KB, Nelson SM, Dosenbach NU, Schlaggar BL, Mumford JA, Poldrack RA, Petersen SE, 2015. Functional system and areal organization of a highly sampled individual human brain. Neuron 87 (3), 657–670. 26212711 10.1016/j.neuron.2015.06.037 PMC4642864

[R31] Menon V, 2011. Large-scale brain networks and psychopathology: a unifying triple network model. Trends Cogn Sci 15 (10), 483–506. 21908230 10.1016/j.tics.2011.08.003

[R32] Moreno-Fernandez J, Ochoa JJ, Lopez-Frias M, Diaz-Castro J, 2020. Impact of early nutrition, physical activity and sleep on the fetal programming of disease in the pregnancy: a narrative review. Nutrients 12 (12), 3900. 10.3390/nu12123900 PMC7766505 33419354

[R33] Ng KK, Lo JC, Lim JKW, Chee MWL, Zhou J, 2016. Reduced functional segregation between the default mode network and the executive control network in healthy older adults: a longitudinal study. Neuroimage 133, 321–330. 27001500 10.1016/j.neuroimage.2016.03.029

[R34] Onoda K, Ishihara M, Yamaguchi S, 2012. Decreased functional connectivity by aging is associated with cognitive decline. J Cogn Neurosci 24 (11), 2186–2198. 22784277 10.1162/jocn_a_00269

[R35] Pardo JV, Lee JT, Sheikh SA, Surerus-Johnson C, Shah H, Munch KR, Carlis JV, Lewis SM, Kuskowski MA, Dysken MW, 2007. Where the brain grows old: decline in anterior cingulate and medial prefrontal function with normal aging. Neuroimage 35 (3), 1231–1237. 17321756 10.1016/j.neuroimage.2006.12.044 PMC1913629

[R36] Radhakrishnan R, Elsaid NMH, Sadhasivam S, Reher TA, Hines AC, Yoder KK, Saykin AJ, Wu YC, 2021. Resting state functional MRI in infants with prenatal opioid exposure-a pilot study. Neuroradiology 63 (4), 585–591. 32978671 10.1007/s00234-020-02552-3 PMC9162800

[R37] Reiman EM, Caselli RJ, Yun LS, Chen K, Bandy D, Minoshima S, Thibodeau SN, Osborne D, 1996. Preclinical evidence of Alzheimer’s disease in persons homozygous for the epsilon 4 allele for apolipoprotein E. N Engl J Med 334 (12), 752–758. 8592548 10.1056/NEJM199603213341202

[R38] Roos A, Fouche JP, Ipser JC, Narr KL, Woods RP, Zar HJ, Stein DJ, Donald KA, 2020. Structural and functional brain network alterations in prenatal alcohol exposed neonates. Brain Imaging Behav 15 (2), 689–699. doi:10.1007/s11682-020-00277-8. PMC7572489 32306280

[R39] Salzwedel AP, Grewen KM, Vachet C, Gerig G, Lin W, Gao W, 2015. Prenatal drug exposure affects neonatal brain functional connectivity. J Neurosci 35 (14), 5860–5869. 25855194 10.1523/JNEUROSCI.4333-14.2015 PMC4388938

[R40] Scheinost D, Kwon SH, Lacadie C, Sze G, Sinha R, Constable RT, Ment LR, 2016. Prenatal stress alters amygdala functional connectivity in preterm neonates. Neuroimage Clin 12, 381–388. 27622134 10.1016/j.nicl.2016.08.010 PMC5009231

[R41] Silverman DH, Small GW, Chang CY, Lu CS, Kung De Aburto MA, Chen W, Czernin J, Rapoport SI, Pietrini P, Alexander GE, Schapiro MB, Jagust WJ, Hoffman JM, Welsh-Bohmer KA, Alavi A, Clark CM, Salmon E, de Leon MJ, Mielke R, Cummings JL, Kowell AP, Gambhir SS, Hoh CK, Phelps ME, 2001. Positron emission tomography in evaluation of dementia: regional brain metabolism and long-term outcome. JAMA 286 (17), 2120–2127. 11694153 10.1001/jama.286.17.2120

[R42] Thomason ME, 2020. Development of brain networks in utero: relevance for common neural disorders. Biol Psychiatry 88 (1), 40–50. 32305217 10.1016/j.biopsych.2020.02.007 PMC7808399

[R43] Thomason ME, Grove LE, Lozon TA Jr., Vila AM, Ye Y, Nye MJ, Manning JH, Pappas A, Hernandez-Andrade E, Yeo L, Mody S, Berman S, Hassan SS, Romero R, 2015. Age-related increases in long-range connectivity in fetal functional neural connectivity networks in utero. Dev Cogn Neurosci 11, 96–104. 25284273 10.1016/j.dcn.2014.09.001 PMC4532276

[R44] Thomason ME, Hect JL, Rauh VA, Trentacosta C, Wheelock MD, Eggebrecht AT, Espinoza-Heredia C, Burt SA, 2019. Prenatal lead exposure impacts cross-hemispheric and long-range connectivity in the human fetal brain. Neuroimage 191, 186–192. 30739062 10.1016/j.neuroimage.2019.02.017 PMC6451829

[R45] Woo CW, Krishnan A, Wager TD, 2014. Cluster-extent based thresholding in fMRI analyses: pitfalls and recommendations. Neuroimage 91, 412–419. 24412399 10.1016/j.neuroimage.2013.12.058 PMC4214144

[R46] Wozniak JR, Mueller BA, Muetzel RL, Bell CJ, Hoecker HL, Nelson ML, Chang PN, Lim KO, 2011. Inter-hemispheric functional connectivity disruption in children with prenatal alcohol exposure. Alcohol Clin Exp Res 35 (5), 849–861. 21303384 10.1111/j.1530-0277.2010.01415.x PMC3083458

