# Supplementary material for: Sex-specific effects of prenatal undernutrition on resting-state functional connectivity in the human brain at age 68
Source: Neurobiol Aging. Author manuscript; Available in PMC 2022 Sep 9. (PMC9459445; doi:10.1016/j.neurobiolaging.2022.01.003)
Supplement: supp [file NIHMS1831825-supplement-supp.docx]

# 6 Supplement

## 6.1 Supplementary materials

### 6.1.1 Study parameters

Maternal and birth characteristics were available from medical birth records. A standardized interview was completed during the home visit by a trained research assistant providing detailed information on adult characteristics. Educational level was measured on a 10-point scale (1=primary education not completed, 10=university completed). Socioeconomic status (SES) was derived from occupational status of the participant’s or their partner’s occupation (whichever was highest), according to the standard international socioeconomic index of occupational status (ISEI-92). Smoking was defined as ever having smoked. In addition, information was obtained on hypertension, hypercholesterolemia, diabetes and stroke or transient ischemic attack (TIA, ever diagnosed), and the hospital anxiety and depression scale (HADS) was completed. Total brain volume (TBV, excluding ventricles) and BrainAGE scores were derived from structural MRI scans collected during the same scan session, as reported previously (de Rooij et al., 2016; Franke et al., 2018).

### 6.1.2 Seed regions of interest

In this network atlas, the DMN consists of four subregions (medial prefrontal cortex (MPFC), left and right lateral parietal (LP) and precuneus cortex (PCC)), the SN of seven subregions (anterior cingulate cortex (ACC), left and right anterior insula (AI), left and right rostral prefrontal cortex (RPFC), left and right supramarginal gyrus (SMG)) and the CEN of four subregions (left and right lateral prefrontal cortex (LPFC), left and right posterior parietal cortex (PPC)). A visual representation of these network ROIs is depicted in Supplementary Fig. 1.

### 6.1.3 MRI data preprocessing

Raw images were visually inspected for abnormalities and artifacts by a researcher. In case of abnormal findings, scans were examined by a radiologist. Participant scans were excluded based on excessive motion defined as a mean absolute displacement of >2.3 mm.

Image preprocessing was performed in Statistical Parametric Mapping (SPM, version 12, <https://www.fil.ion.ucl.ac.uk/spm/>) and included realignment to a subject-specific high-resolution T1-weighted image obtained during the same scan session. Data were spatially normalized to the Montreal Neurological Institute (MNI) template and smoothed using a 6-mm Gaussian kernel.

We performed further image processing and analysis using the CONN toolbox (version 2018b, <http://nitrc.org/projects/conn>) (Whitfield-Gabrieli and Nieto-Castanon, 2012). The default denoising pipeline implemented in CONN was used for noise correction. Briefly, the CONN denoising pipeline performs linear regression of potential confounding effects in the BOLD signal using the anatomical component-based noise correction procedure aCompCor (Behzadi et al., 2007). The potential confounding effects included components from white matter and cerebrospinal areas from the subject’s own anatomical segmentation, estimated subject-motion parameters and constant and first-order linear session effects (Friston et al., 1996; Whitfield-Gabrieli and Nieto-Castanon, 2012). A temporal bandpass filter of 0.008-0.09 Hz was applied to focus on low-frequency fluctuations whilst limiting noise effects.

## 6.2 Supplementary Tables

**Supplementary Table 1 Significant within-network group effects across all participants using smoothed data to extract the ROI BOLD time series**

| **Network** | **Seed region** | | **Subregion effect** | **Direction of effect** | **FWE cluster threshold at p<0.01** | **Cluster size at p<0.01** | **FWE cluster threshold at p<0.05** | **Cluster size at p<0.05** |
| --- | --- | --- | --- | --- | --- | --- | --- | --- |
| CEN | | PPC L | LPFC L | EE > HC | 46 | 40 | 134 | 122 |
| DMN | | MPFC | LP R | HC > EE | 103 | NA | 290 | NA |
| DMN | | LP L | MPFC | HC > EE | 107 | 3 | 314 | 263^a^ |
| SN | | SMG R | RPFC R | HC > EE | 80 | 33 | 205 | 140 |

PPC = posterior parietal cortex, LPFC = lateral prefrontal cortex, MPFC = medial prefrontal cortex, LP = lateral parietal, SMG = supramarginal gyrus, RPFC = rostral prefrontal cortex, L = left, R = right, HC = healthy controls, EE = early exposed, FWE = Family-Wise Error.

^a^Additional significant cluster present within PCC subregion

**Supplementary Table 2 Sex-specific significant within-network group effects using smoothed data to extract the ROI BOLD time series**

| **Network** | **Seed region** | **Subregion effect** | **Direction of effect** | **FWE cluster threshold at p<0.01** | **Cluster size at p<0.01** | **FWE cluster threshold at p<0.05** | **Cluster size at p<0.05** |
| --- | --- | --- | --- | --- | --- | --- | --- |
| **Men** | | | |  |  |  |  |
| DMN | LP L | PCC | EE > HC | 72 | 55 | 206 | 403 |
| SN | RPFC L | ACC | EE > HC | 43 | 26 | 136 | 96 |
| SN | AI L | ACC | EE > HC | 39 | 3 | 99 | 56 |
| SN | AI L | RPFC L | EE > HC | 39 | NA | 99 | 4 |
| CEN | PPC R | PPC L | EE > HC | 46 | 3 | 114 | 148 |
| DMN | LP L | MPFC | HC > EE | 72 | 30 | 206 | 350 |
| SN | AI L | SMG L | HC > EE | 39 | 23 | 99 | 93 |
| **Women** | | | |  |  |  |  |
| CEN | LPFC L | LPFC R | EE > HC | 62 | 12 | 200 | 235 |
| CEN | LPFC L | PPC R | EE > HC | 62 | 22 | 200 | 105 |
| CEN | PPC La | LPFC L | EE > HC | 32 | 49, 5 | 80 | 283 |
| CEN | PPC L | LPFC R | EE > HC | 32 | 5 | 80 | 47 |
| CEN | LPFC R | LPFC L | EE > HC | 54 | 79 | 139 | 221 |
| DMN | LP R | MPFC | HC > EE | 80 | 50 | 255 | 363 |
| SN | AI L | RPFC R | HC > EE | 59 | 29 | 165 | 137 |
| SN | RPFC R | AI L | HC > EE | 95 | 92 | 273 | 169 |
| SN | SMG L | RPFC R | HC > EE | 54 | 57 | 135 | 258 |
| CEN | PPC L | LPFC R | HC > EE | 32 | 21 | 80 | 113 |
| CEN | PPC L | PPC R | HC > EE | 32 | NA | 80 | 71 |

LP = lateral parietal, PCC = precuneus cortex, RPFC = rostral prefrontal cortex, ACC = anterior cingulate cortex, AI = anterior insula, SMG = supramarginal gyrus, PPC = posterior parietal cortex, MPFC = medial prefrontal cortex, L = left, R = right, HC = healthy controls, EE = early exposed, FWE = Family-Wise Error. NA = cluster not present.

^a^Two significant clusters were identified between these regions.

## 6.3 Supplementary Figures


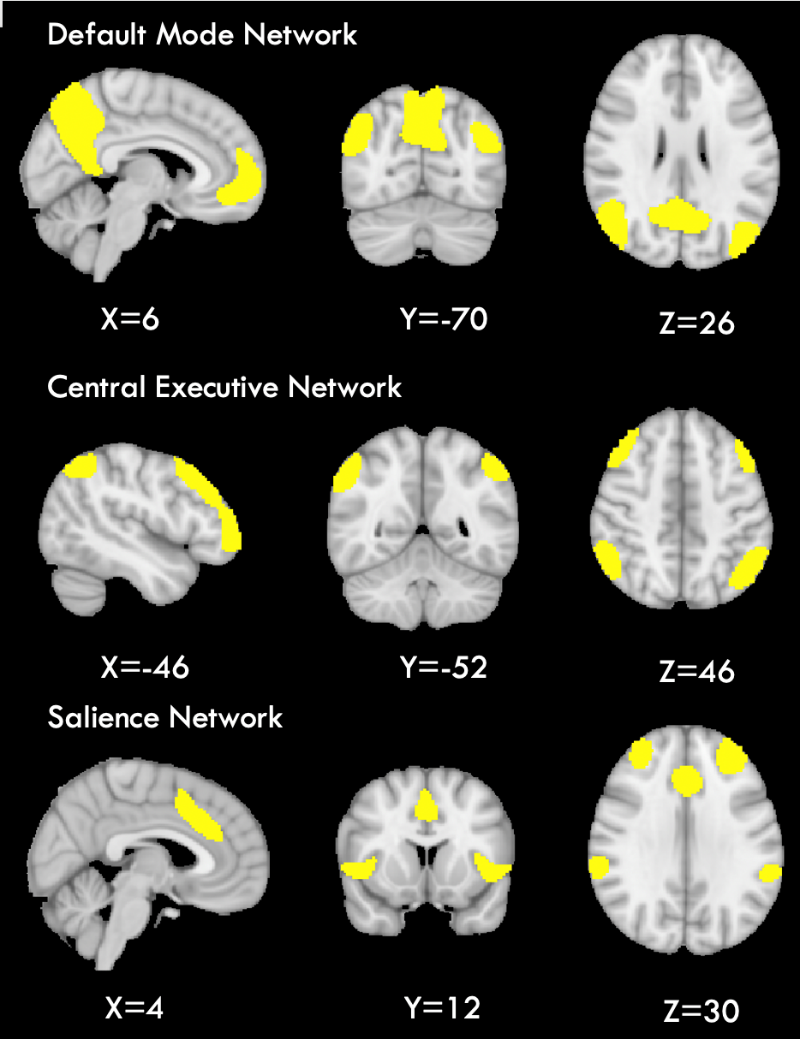


**Supplementary Figure 1 Seed regions of interest.** Coronal, sagittal and axial view of the binary CONN network ROIs of the DMN, CEN and SN, overlaid on the MNI 152 brain. The ROIs were created by CONN’s ICA analyses of the HCP dataset.


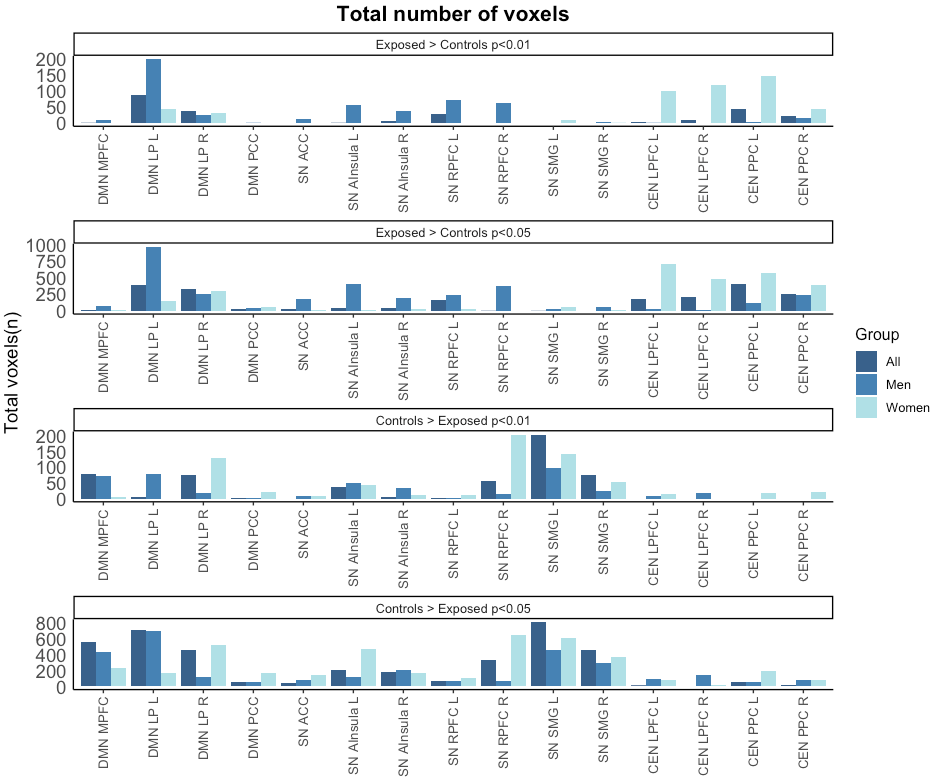


**Supplementary Figure 2 Total number of voxels.** The total number of significant voxels present within the mask at a voxel level primary threshold of p < 0.01 and p < 0.05 for each ROI masked with the combined map of the other ROIs in the network.


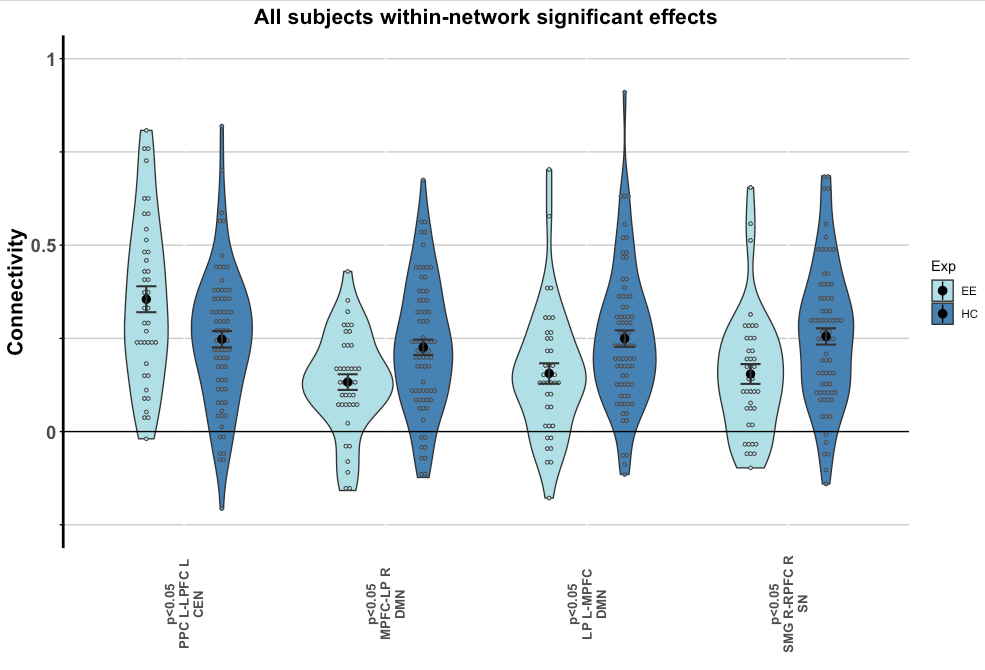


**Supplementary Figure 3 Mean within-network connectivity values across all participants.** Mean connectivity values of all significant within-network effects across all participants.


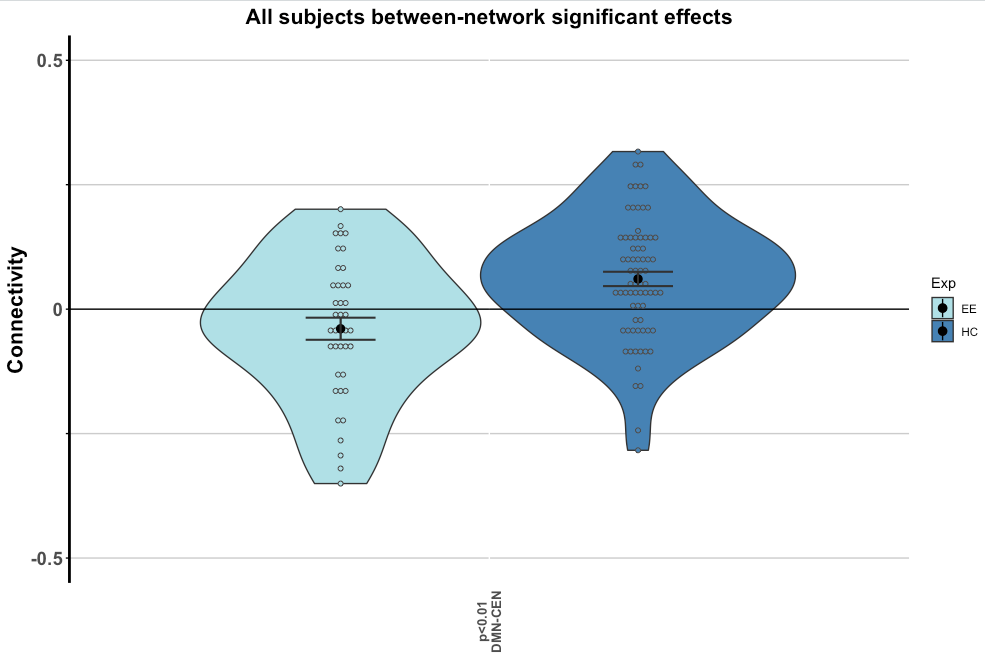


**Supplementary Figure 4 Mean between-network connectivity values across all participants.** Mean connectivity values of all significant between-network effects across all participants
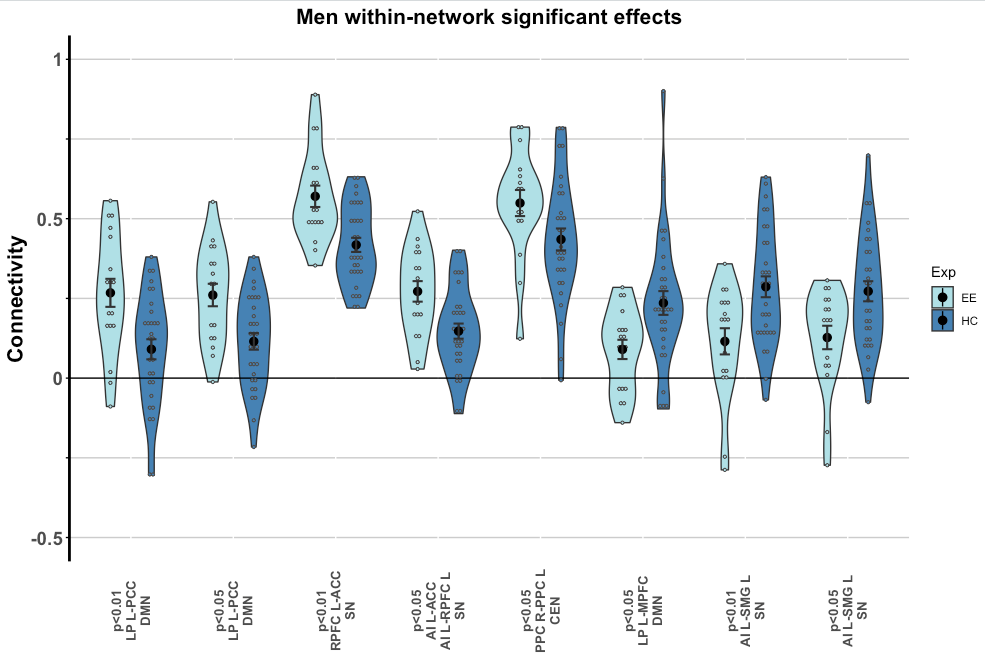


**Supplementary Figure 5 Mean within-network connectivity values in men**. Mean connectivity values of all significant within-network effects in men.


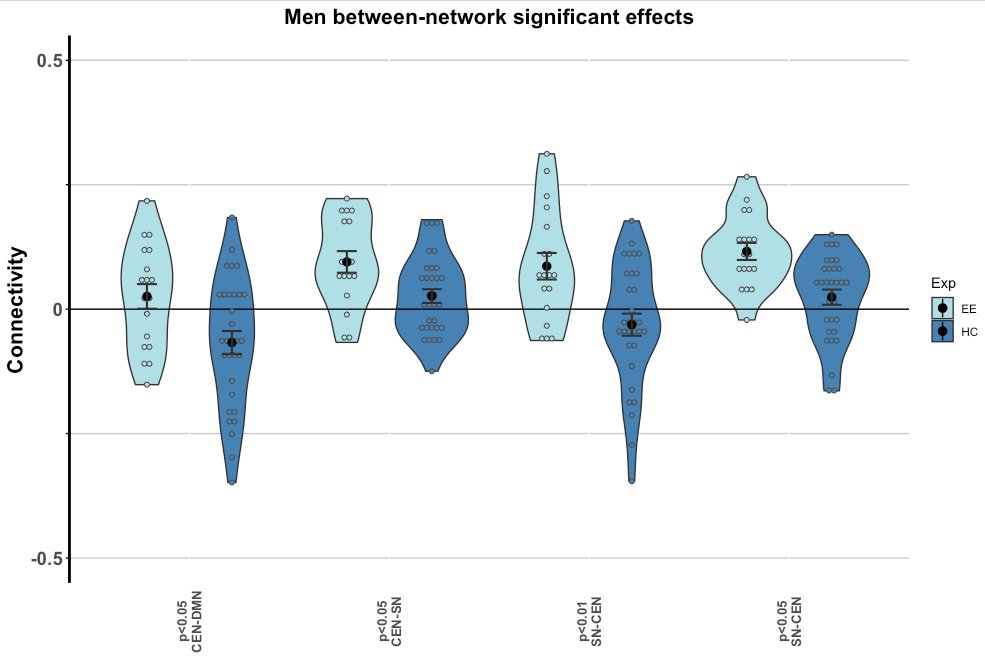


**Supplementary Figure 6 Mean between-network connectivity values in men.** Mean connectivity values of all significant between-network effects in men.


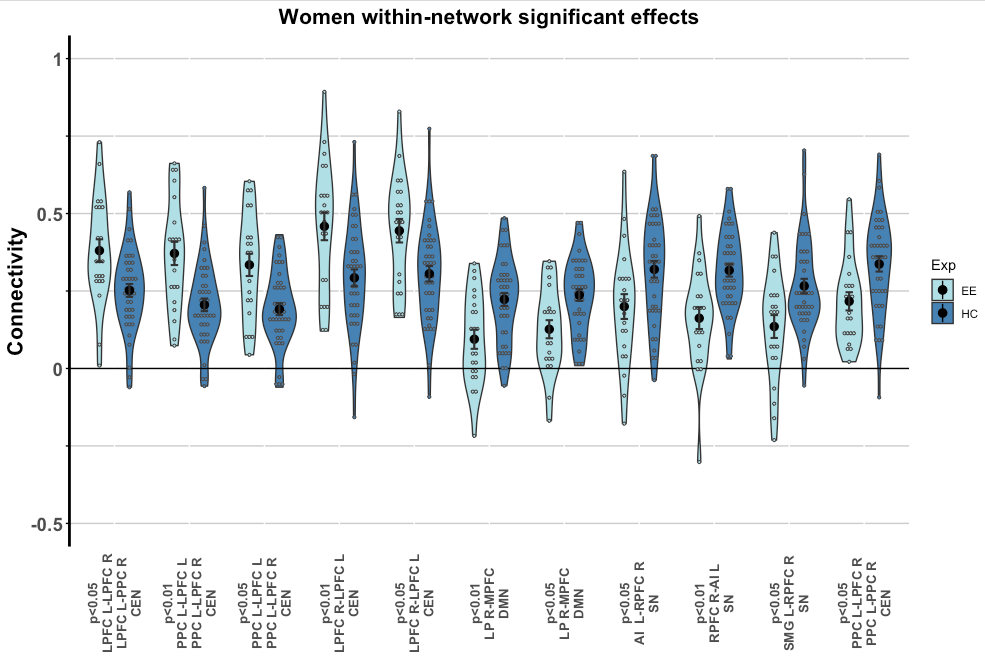


**Supplementary Figure 7** **Mean within-network connectivity values in women**. Mean connectivity values of all significant within-network effects in women.


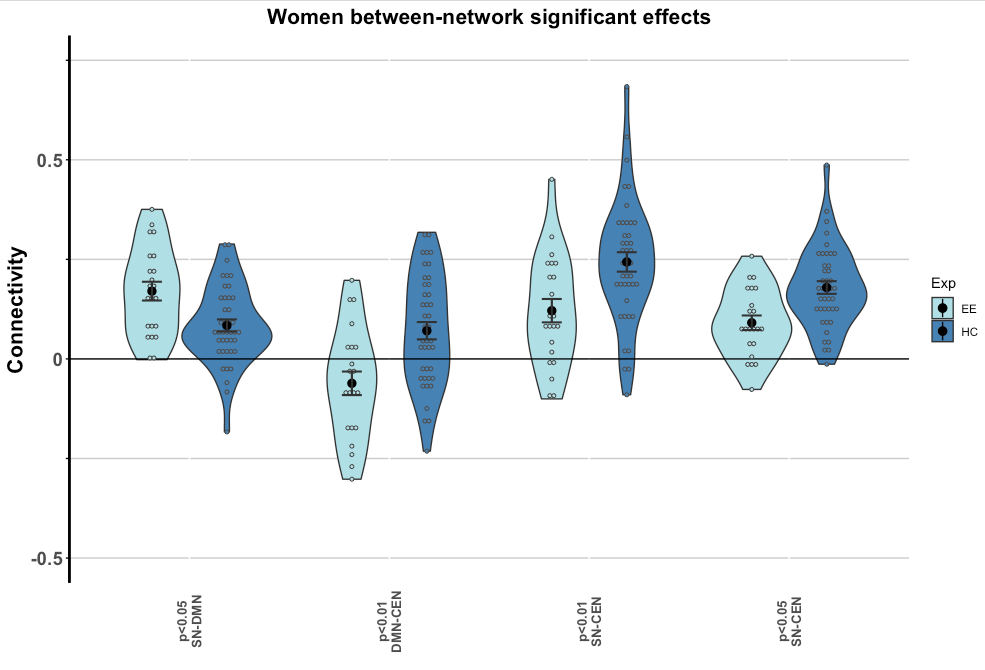


**Supplementary Figure 8 Mean between-network connectivity values in men.** Mean connectivity values of all significant between-network effects in women.

## 6.4 Supplement References

1. Behzadi, Y., Restom, K., Liau, J., Liu, T.T., 2007. A component based noise correction method (CompCor) for BOLD and perfusion based fMRI. Neuroimage 37(1), 90-101.

2. de Rooij, S.R., Caan, M.W., Swaab, D.F., Nederveen, A.J., Majoie, C.B., Schwab, M., Painter, R.C., Roseboom, T.J., 2016. Prenatal famine exposure has sex-specific effects on brain size. Brain 139(Pt 8), 2136-2142.

3. Franke, K., Gaser, C., Roseboom, T.J., Schwab, M., de Rooij, S.R., 2018. Premature brain aging in humans exposed to maternal nutrient restriction during early gestation. Neuroimage 173, 460-471.

4. Friston, K.J., Williams, S., Howard, R., Frackowiak, R.S., Turner, R., 1996. Movement-related effects in fMRI time-series. Magn Reson Med 35(3), 346-355.

5. Whitfield-Gabrieli, S., Nieto-Castanon, A., 2012. Conn: a functional connectivity toolbox for correlated and anticorrelated brain networks. Brain Connect 2(3), 125-141.
